# Psychological Factors Explaining Perceived Impact of COVID-19 on Travel

**DOI:** 10.3390/ejihpe11040083

**Published:** 2021-09-24

**Authors:** José Magano, Diogo Guedes Vidal, Hélder Fernando Pedrosa e Sousa, Maria Alzira Pimenta Dinis, Ângela Leite

**Affiliations:** 1 Research Center in Business and Economics (CICEE), Universidade Autónoma de Lisboa, Rua Sta. Marta 47, 5° Andar, 1150-293 Lisboa, Portugal; 2 ISCET—Higher Institute of Business Sciences and Tourism, Rua de Cedofeita, 285, 4050-180 Porto, Portugal; 3 UFP Energy, Environment and Health Research Unit (FP-ENAS), University Fernando Pessoa (UFP), Praça 9 de Abril 349, 4249-004 Porto, Portugal; diogovidal@ufp.edu.pt; 4 Department of Mathematics (DM), University of Trás-os-Montes and Alto Douro (UTAD), Quinta de Prados, 5001-801 Vila Real, Portugal; hfps@utad.pt; 5 School of Human and Social Sciences (ECHS), University of Trás-os-Montes and Alto Douro (UTAD), Quinta de Prados, 5001-801 Vila Real, Portugal; angelal@utad.pt

**Keywords:** COVID-19, anxiety, stress, travel, wellness

## Abstract

This cross-sectional study aims to determine the psychological factors that contribute to the perceived impact of COVID-19 on travel using a convenience sample (*N* = 1122) from the general population to whom instruments assessing the perception of the COVID-19 pandemic’s impact on travel, anxiety, fear, phobia, risk perception, and stress were applied. The participants were mainly female (65.6%), had not attended university, and who were professionally active, with a mean age of around 30-years-old (*M* = 31.91, *SD* = 13.76, *Min* = 18, *Max* = 81). The perceived impact of COVID-19 on travel correlates with all of the psychological variables, mainly in terms of the emotional fear of COVID-19. Together with the perceived risk of COVID-19, social phobia due to COVID-19, and COVID-19 stress contamination, these variables explain 20% of the perceived impact of COVID-19 on travel variance. The relationship between COVID-19 stress socio-economic consequences and the perception of the pandemic’s impact on travel is moderated by the emotional perceived risk of COVID-19. Fear and perception of this risk explain the impact of the COVID-19 on travel in pandemic times, suggesting that the psychological impact of fear and anxiety induced by the pandemic needs to be handled as a public health priority.

## 1. Introduction

The economic and social impacts of the pandemic on travel are portrayed by the mass media daily. In fact, according to Škare et al. [1], the pandemic has had a destructive impact on the travel and tourism industry. For Lee and Chen [2], the crisis’ impacts on the travel and leisure industry are undeniable. Abdullah et al. [3] stated that significant changes in the primary purposes for traveling have been observed due to the pandemic, namely people tending to use less public transport and more private cars; there was a modal shift to active modes of transportation from public transport and paratransit that was significant; and people placed more priority on pandemic related factors when choosing a mode of transportation. Additionally, Barbieri et al. [4] found great disruptions for both commuting and non-commuting travel and great reductions in the frequency of all types of trips and the use of all available modes of transportation. These changes in travel patterns could result in economic and social implications, the extent of which cannot yet be fully realized.

However, the media does not pay the same attention to the psychological impact of the COVID-19 pandemic, which has a negative effect on economic activities, and example of which is people’s desire to travel. In fact, the circumstances surrounding the COVID-19 pandemic affect mental well-being [5,6] and quality of life (QoL) [7], with QOL being related to personal well-being and includes aspects such as health, leisure, personal satisfaction, habits, and lifestyle [8]. Studies assessing mental health in the pandemic context have shown that individuals report high levels of anxiety [9], depression [10,11], fear [12], stress [13], risk perception [14], and phobia [15]. In fact, according to Taylor et al. [13], during a pandemic, many people present stress or anxiety, specifically the fear of becoming infected, the fear of coming into contact with possibly contaminated objects or surfaces, the fear of foreigners who might be carrying infection (disease-related xenophobia), and the fear of socio-economic consequences. Moreover, gender differences in terms of health risks and implications are likely to be expanded during the COVID-19 pandemic [16]. However, few studies exist on how these issues affect tourist activity, and the studies that do exist on the subject mainly focus on risk perception. Specifically, Neuburger and Egger [17] found that the risk perception of COVID-19, travel risk perception, and the willingness to change or cancel travel plans has significantly increased. The perception of health risks is negatively related to the perception of a destination’s safety and can influence travel intentions. Risk aversion is higher when tourists consider international tourism versus domestic tourism [18]. Moreover, the risk perceived by the respondents has a negative influence on their attitude towards traveling [19]. Threat severity and susceptibility can cause “travel fear”, which has led to protective travel behaviors during the pandemic [20]. The perceived threat of the pandemic generates uncertainty and fear [21]. COVID-19 may trigger several fears (e.g., contamination, future, financial instability, xenophobia, and agoraphobia) and may trigger elements related to anxiety and fear (similar to specific phobias) [9]. Additionally, having a fear of COVID-19 has been shown to negatively impact travel [22]. However, these studies were dedicated to studying isolated psychological variables and not to seeking an explanatory model for the impact of the pandemic on travel from a constellation of psychological variables that have been previously studied outside of the pandemic context.

Accordingly, several authors have felt the need to develop adequate instruments to measure psychological aspects that are specifically related to COVID-19, namely anxiety [9], fear [12], risk perception [14], phobia [15], and stress [13]. However, there are no investigations in which these variables are studied together in an attempt to find an explanatory model. The research question of this study is whether the psychological impact of the pandemic, i.e., anxiety, fear, phobia, stress, and risk perception, affects traveling. This issue is very important, as there are unrealistic expectations that travel- and tourism-related economic activity will be improve once government-imposed restrictions are lifted. Although this is partially true, the psychological impact of the COVID-19 pandemic may prevent such a rebound from actually happening.

Given the current nature of the COVID-19 pandemic and the few studies that have been conducted on the impact of the pandemic on travel, very little is known about the psychological variables that factor into this equation and that explain the perceived impact of COVID-19 on travel. Based on the previous studies mentioned above and based on the fact that the aforementioned variables can be assessed using validated instruments, it has been hypothesized that these psychological factors that have been studied in the literature, i.e., anxiety of COVID-19, fear of COVID-19, perceived risk of COVID-19, COVID-19 phobia, and COVID-19 stress, contribute the explanation of the impact of COVID-19 on travel. Consequently, this study aims to determine the psychological factors that contribute to the perceived impact of COVID-19 on travel.

## 2. Materials and Methods

### 2.1. Procedures

The data collection protocol was approved by the University of Trás-os-Montes Institutional Research Ethics Board. The study was published on a social media page. After reading the objectives and purposes and signing informed consent, the participants could complete the questionnaire. The instruments that were used were validated for the Portuguese population. Data were collected online through *Survey Monkey* between 1 October and 15 November 2020. This was a random and non-representative sample of the Portuguese population, having the inclusion criteria of being over 18 years of age and being of Portuguese nationality.

### 2.2. Measures

In addition to questions regarding sociodemographics (gender, age, education, and employment status), the protocol used in this study included seven questions concerning the perceived impact of COVID-19 on travel, four psychological instruments previously validated to the Portuguese population related to COVID-19 (COVID-19 anxiety scale; fear of COVID-19 scale; COVID-19 Perceived Risk Scale and COVID-19 Phobia), and 24 items of the COVID-19 stress scales [13].

#### 2.2.1. Perceived Impact of COVID-19 on Travel

Seven questions assessed the participants’ perceptions of the impact of COVID-19 on travel during the COVID-19 pandemic. The instruction (“On a scale of 0 to 100, please indicate how much the pandemic situation caused by COVID-19 has…”) preceded the items: 1—… “changed your leisure activities”; 2—… “changed your vacation”; 3—… “prevented you from settling in a hotel”; 4—… “prevented you from traveling by plane”; 5—… “prevented you from traveling by train”; 6—… “prevented you from traveling by car”; 7—… “made you feel afraid to frequent hotel facilities”. These issues were analyzed individually as well as in a single construct.

#### 2.2.2. COVID-19 Anxiety Scale (Portuguese Version)

According to Lee [23], the CAS is a brief mental health screener that can identify cases of dysfunctional anxiety and symptom severity associated with the coronavirus. It is a unidimensional five-item scale that assesses the physiological reactions of anxiety related to COVID-19. The original version is highly reliable as a cluster (α = 0.93), according to Lee [2,23]. Additionally, the Portuguese version presents a Cronbach’s alpha of 0.85 [22].

#### 2.2.3. Fear of COVID-19 Scale (Portuguese Version)

The FC19S is a unidimensional seven-item scale. According to Ahorsu et al. [12], the FC19S is reliable and valid in assessing the fear of COVID-19 among the general population and is useful in allaying COVID-19 fears among individuals. For the original version, Cronbach’s alpha is 0.82, whereas in the Portuguese version, it is 0.88. However, in the Portuguese version, the authors found that two factors had a different structure: emotional fear (items 1,2,4,5; α = 0.83) and cognitive fear (items 3,6,7; α = 0.82) [22].

#### 2.2.4. COVID-19 Perceived Risk Scale (Portuguese Version)

Yıldırım and Güler [14] conceived the COVID-19 perceived risk scale by adapting the eight-item Severe Acute Respiratory Syndrome (SARS) Risk Perception Scale [24] and by changing the wording of the original items. The scale includes a cognitive and an emotional dimension of personal risk. High scores indicate high levels of perceived risk related to COVID-19. In the original version, reliability ranged from 0.70 to 0.74 for the cognitive dimension and from 0.84 to 0.88 for the emotional dimension, suggesting satisfactory internal consistency reliability for the C19PRS. In the Portuguese version, reliability was 0.68 for the cognitive dimension, 0.85 for the emotional dimension, and 0.80 for the full assessment [15].

#### 2.2.5. COVID-19 Phobia Scale (C19PS; Portuguese Version)

Arpaci et al. [25] developed this scale to detect COVID-19 phobia early on in order to to intervene as soon as possible. The authors assumed that the development of this phobia is due to the psychological burden caused by COVID-19. They found a structure of 20 items and 4 subscales (psychological, psycho-somatic, economic, and social factors). In the original version, Arpaci et al. [25] found that the reliability ranged from 0.85 to 0.90 for the four factors and was 0.92 for the full assessment, suggesting good internal consistency reliability for the C19PS. In the Portuguese version, the authors maintained the original structure of the scale and found a Cronbach’s alpha of 0.92 for the full assessment; for all of the other subscales, the reliability ranged from 0.73 to 0.91 [15].

#### 2.2.6. COVID Stress Scales

Taylor et al. [13] developed the 36-item COVID-19 Stress Scale (CSS), which consists of 36 items to better understand and assess COVID-19-related distress. A 5-factor solution was identified: (1) danger and contamination fears; (2) fears about economic consequences; (3) xenophobia; (4) compulsive checking and reassurance-seeking; and (5) traumatic stress symptoms regarding COVID-19. Reliability ranged from 0.83 to 0.95. The scales were intercorrelated, providing evidence of COVID-19 Stress Syndrome [13]. This study only used the first 24 items of the scale that corresponded to the first four subscales. This option was mainly due to the fact that the scale was extensive, and the authors were particularly interested in the stress associated with the danger, contamination, fear of economic consequences, and the xenophobia subscales. A confirmatory factor analysis was conducted to assess the adequacy of the model to the data (*χ*^2^ (*df*) = 4.1; CFI = 0.962; TLI = 0.955; RMSEA = 0.053; PCLOSE = 0.081; SRMR = 0.064). All of the indicators were within the expected values (*χ*^2^ (*df*) < 2, CFI and TLI > 0.95; RMSEA < 0.07; PCLOSE > 0.05; SRMR < 0.08), with the exception of *χ*^2^, which was greater than 2. However, large samples, in cases such as this, can cause an increase in the value of *χ*^2^ [26]. In this study, the alpha values for all of the dimensions that were used in the present work (danger stress, socio-economic consequences stress, xenophobia stress, and contamination stress) were 0.90.

### 2.3. Data Analysis

Descriptive statistics were used to describe the sample and the instruments (frequencies, percentages, means, standard deviations, ranges); skewness and kurtosis assessed the normality of the distribution of the variables. Inferential statistics were used to draw conclusions from the sample and to generalize them to a population (Student’s *t*-test, chi-square test, Pearson correlations, multiple linear regression, and moderation). Student’s *t*-test was used to determine if there is a significant difference between the means of two groups. The chi-square test was used to determine whether there was a statistically significant difference between the expected frequencies and the observed frequencies in one or more categories of a contingency table. Multiple regression models were used to determine how a single response variable *Y* depends linearly on a number of predictor variables. Moderation was used to determine if the strength and the direction of the relationship between two variables depend on a third variable, with this third variable being the moderator. The value of the statistical power for the *t*-test was 1, with the critical value being1.6; for the chi-square test it was 1, with the critical value being 16.9; for multiple regression it was 1, with the critical value being 2.1 [27]. Cronbach’s alpha coefficient assessed reliability as internal consistency. Correlational analyses were conducted based on the significance of correlations, as suggested by Cohen [28]: *r* = 0.1 (small), 0.3 (moderate), 0.5 (large).

### 2.4. Participants

A total of 1122 people participated in this study, most of whom were (n = 725/64.6%), with a mean age of 31.9 years of age (SD = 13.8; Min = 18 − Max = 81). Most of the participants had not previously studied at university (n = 627/55.9%) and were professionally active (n = 932/83.1%) (Table 1).

## 3. Results

Table 1 also presents the perceived impact of COVID-19 on travel items concerning the total sample as well as the value of Cronbach’s alpha to assess the reliability of the total perceived impact of COVID-19 on travel. It also displays the average values of the same variables according to gender and the significance of their differences. The skewness and kurtosi values ensured the normal distribution of the variables (reference values of |*sk*| < 3 and |*ku*| < 11) [29]. Most of the sample was female (65.6%),, had not studied at university, were professionally active, and had a mean age of around 30-years-old (*M* = 31.9, *SD* = 13.8, *Min* = 18, *Max* = 81). There were no statistically significant differences between the men and women involved in the study concerning age, education, and employment status. The impact of COVID-19 on the use of a car to travel was the issue that scored the lowest, whereas issues related to the impact of the pandemic on leisure and vacation scored the highest. There were statistically significant differences between the men and women participating in the study concerning all of the items and in total, with the exception of the question regarding the impact of the pandemic on the car use. Women scored higher than men on all of items and in total.

There were also statistically significant differences between the participants had previously attended university and those who had not concerning issues related to vacation (*t*(1107, 17) = −3.5; *p* < 0.001; *d* = −0.2), traveling by plane (*t*(1097, 92) = −4.8; *p* < 0.001; *d* = −0.3), traveling by train (*t*(1120) = −5.2; *p* < 0.001; *d* = −0.3), and the total perceived impact of COVID-19 on travel (*t*(1120) = −4.1; *p* < 0.001; *d* = −0.3). People with higher education scored above those without higher education in all of these dimensions. With regard to the employment situation, statistically significant differences were only found in terms of the use of hotel equipment (*t*(1120) = 2.1; *p* = 0.03; *d* = 0.8), showing that inactive people have significantly higher values than active people. There were significant and positive correlations between age and issues related to traveling by plane (*r* = 0.1; *p* = 0.002), traveling by train (*r* = 0.1; *p* = 0.001), and traveling by car (*r* = 0.1; *p* = 0.004). However, the values of these correlations are very weak. Older people tended to score higher on these issues than younger people.

Table 2 shows the descriptive statistics of the psychological variables concerning the total sample and the values of Cronbach’s alpha. It also presents the average values of the same variables according to gender and the significance of their differences. The skewness and kurtosis values ensure the normal distribution of the psychological variables, although the value of the COVID-19 anxiety scale is at the limit of the reference values (values of |*sk*| < 3 and |*ku*| < 11) [29]. Internal consistency was assessed through Cronbach’s alpha; all of the variables present values above the recommended (≥0.7) [30]. Regarding the differences in the average values of psychological variables according to gender, the participants of the female gender demonstrated significantly higher values than the participants of the male gender.

Participants who had not attended university showed higher values than participants who had attended university in the following dimensions: COVID-19 fear scale—cognitive fear; COVID-19 phobia scale—total, psychological factors, psychosomatic factors, and economic factors; socio-economic consequences stress; xenophobia stress; and contamination stress. Inactive participants demonstrated higher values than active participants did in the following dimensions: COVID-19 fear scale—cognitive fear; COVID-19 phobia scale—total and economic factors; COVID-19 stress—socio-economic consequences stress; xenophobia stress; and contamination stress (Table 3).

All psychological variables correlate positively and significantly with each other. Correlations between the psychological variables ranged from *r* = 0.9 (stress xenophobia and perceived risk COVID-19 cognitive scale) and *r* = 0.8 (COVID-19 phobia scale total and fear of COVID-19- scale total) (Table 4). Additionally, the total perceived impact of COVID-19 on travel correlated with all psychological variables, ranging from *r* = 0.2 (perceived risk COVID-19 cognitive scale) to *r* = 0.4 (fear COVID-19—scale emotional fear) (Table 4).

Table 5 displays the results of multiple linear regression for the total perceived impact of COVID-19 on travel. All 16 psychological variables were included in the model because all of them correlated significantly with the total perceived impact of COVID-19 on travel. The four psychological variables that remained in the model were the only ones that significantly contributed to it. The model is statistically significant (*F*(4, 1117) = 70.1; *p* < 0.001) and shows that emotional fear of COVID-19, perceived risk of COVID-19, social phobia of COVID-19, and COVID-19 contamination stress explain 20% of the total perceived impact of COVID-19 on travel variance. Emotional fear of COVID-19 and social phobia caused by COVID-19 were the variables that contributed the most to explaining the perceived impact of COVID-19 on travel.

**Figure 1:**
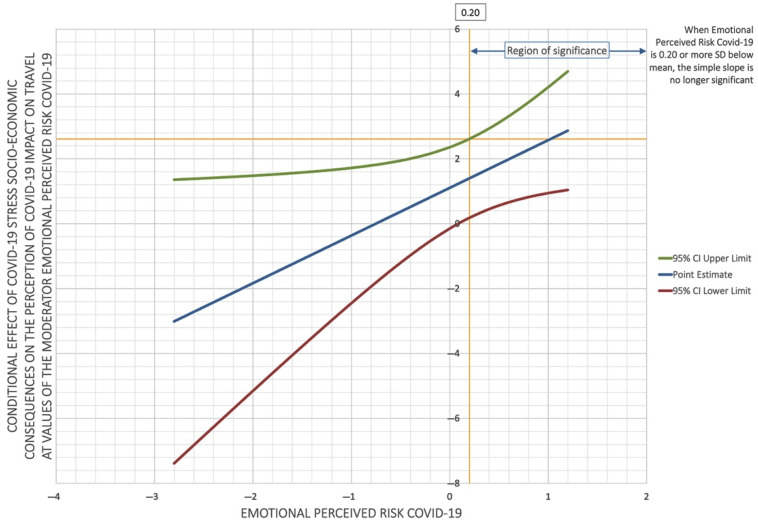
The moderator role of emotional perceived risk.

A moderation analysis was conducted to test the hypothesis that the emotional perceived risk of COVID-19 plays a moderating role between COVID-19 socio-economic stress and the perception of the pandemic’s impact on travel. The PROCESS [31] program was used for this, with the perception of the pandemic’s impact on travel as the dependent variable, COVID-19 socio-economic stress as the independent variable, and the emotional perceived risk of COVID-19 as the moderator. The results show that the emotional perceived risk of COVID-19 is a moderator between COVID-19 socio-economic consequences stress and the perception of the pandemic’s impact on travel. The interaction is significant (*β* = 1.5, 95% CI (0.1; 2.9), *t* = 2.07, *p* = 0.038), indicating the relationship between COVID-19 socio-economic consequences stress and the perception of the pandemic’s impact on travel is moderated by the emotional perceived risk of COVID-19. Specifically, when the emotional perceived risk of COVID-19 is high (*β* = 2.5, 95% CI (0.9; 4.1), *t* = 3.1, *p* = 0.002), there is a significant positive association between COVID-19 socio-economic stress and the perception of the pandemic’s impact on travel, indicating a greater probability of the participants presenting a higher perception of the pandemic’s impact on travel (Figure 1). The Johnson–Neyman technique [32] shows that the relationship between COVID-19 socio-economic stress and the perception of the pandemic’s impact on travel is significant when the emotional perceived risk of COVID-19 is more than 0.2 below the mean.

## 4. Discussion

This study aimed to determine the psychological factors that contribute to the perceived impact of COVID-19 on travel. It was hypothesized that psychological factors contribute decisively to the explanation of the impact of COVID-19 on travel. To test this hypothesis, several psychological instruments that assess anxiety, fear, risk perception, phobia, and stress in relation to COVID-19 as independent variables were used. Additionally, several items related to the perception of the impact of COVID-19 on travel, which were grouped in a single dimension, were used.

The main results found in this study relate to the fact that the total perceived impact of COVID-19 on travel correlates with all of studied psychological variables, mainly with the emotional fear of COVID-19. This dimension, together with the perceived risk of COVID-19, social phobia caused by COVID-19, and COVID-19 contamination stress, explain 20% of the total perceived impact of COVID-19 on travel variance, which confirms our hypothesis. This means that fear (phobia and contamination stress) and the perception of risk explain the impact of COVID-19 on travel i during the pandemic. This is in line with Knowles and Olatunji’s [33] study, which stated that contamination fear did not predict COVID-19 anxiety but predicted safety behaviors in response to COVID-19.

Another interesting finding in this study is that the emotional perceived risk of COVID-19 is a moderator between COVID-19 socio-economic consequences stress and the perception of the pandemic’s impact on travel. Specifically, when the emotional perceived risk of COVID-19 is high, there is a significant positive association between socio-economic consequences stress and the perception of the pandemic’s impact on travel. These results are similar to those found by Iorfa et al. [34], who reported that risk perception mediated the association between COVID-19 knowledge and precautionary behavior.

Other results that were obtained here are also curious, namely results regarding the impact of COVID-19 on the use of a car to travel, which scored the lowest; interestingly, this result seems to contradict those suggested by Abdullah et al. [3], who stated that people tend to use less public transport and more private cars. On the contrary, issues related to the impact of COVID-19 on leisure and vacation scored the highest, which is in line with Lee and Chen’s [2] study, who found that the impacts of the pandemic on the travel and leisure industry are undeniable; however, these impacts may vary across subsectors and businesses such as airlines, restaurants, gambling casinos, and recreational services.

Statistically significant differences were found between men and women concerning all of the items that were evaluated and in total (women scored higher than men in all items and total), except for the question regarding the impact on the use of cars. According to Connor et al. [16], the increased risk of certain negative health outcomes and reduced healthcare access experienced by many women are typically exacerbated during pandemics. This situation may explain why women tended to score higher than men for all of the items.

There are also statistically significant differences between participants who had attended and who had not attended university concerning vacation, traveling by plane, traveling by train, and the total perceived impact of COVID-19 on travel. People who had attended higher education scored higher than those who had not attended higher education, which agrees with what was reported by Xiong et al. [35], who found that more education is associated with greater depressive symptoms and fear in pandemic times.

Concerning employment situation, statistically significant differences were only found regarding the use of hotel equipment, with inactive people demonstrating significantly higher values than active people. These results can be explained by the fact that the inactive participants mostly stayed at home during the pandemic. Conroy et al. [36] found that those who stayed at home experienced worse physical and mental health indicators. This decline in physical and mental health may have led inactive participants to perceive the use of hotel equipment as being more threatening than active participants did. Another possible explanation is that inactive people were more affected by economic issues arising from the pandemic, and this can lead to more depression and therefore to the perception that something is more threatening. There are significant and positive correlations between age and issues related to traveling by plane, traveling by train, and traveling by car. However, the correlation values are very weak. Older people tend to score higher on these issues than younger people, which matches with what has been reported by Zheng et al. [20]. In fact, older people have been more affected by the pandemic, not only physically but also psychologically [37], which may have led them to them perceiving situations as more threatening than younger people do.

Regarding the differences in the mean values of the psychological variables according to gender, the participants of the female gender presented significantly higher values than the male participants did, confirming the results of Coelho et al. [9], Connor et al. [16], Duan and Zhu [5], Paredes et al. [21], Roy et al. [10], Sánchez-Cañizares et al. [19], Škare et al. [1], and Zheng et al. [20]. Participants who had not attended university studies demonstrated higher values than the participants who had attended university did in the following dimensions: the COVID-19 fear scale—cognitive fear; COVID-19 phobia scale—total, psychological factors, psychosomatic factors, and economic factors; socio-economic consequences stress; xenophobia stress; and contamination stress. These results agree with those of Zheng et al. [20]. In addition, these results are in line with those of Spring [38], highlighting the importance of health literacy in relation to COVID-19. Generally, more educated people have more health literacy in general [39]. Finally, the inactive participants had higher values than active participants in the dimensions: the COVID-19 fear scale—cognitive fear; COVID-19 phobia scale–total and economic factors; COVID-19 stress—socio-economic consequences stress; xenophobia stress; and contamination stress, confirming the results obtained by Rahman et al. [40].

## 5. Conclusions

The results found in this study show the effect of psychological variables on the behavior of people in terms of travel in the context of the COVID-19 pandemic. This justifies Serafini and colleagues’ [41] position, which states that the psychological impact of the fear and anxiety induced by the rapid spread of the pandemic needs to be clearly recognized as a public health priority. These results may have important practical implications, as travel promoters must take into account the fears and insecurities arising from the COVID-19 pandemic and develop strategies to secure tourists. Despite this study’s findings, further research into the COVID-19 pandemic crisis should be conducted with a larger and more diverse sample and should include actual destination image perceptions so as to take into account factors such as destination-related factors that are likely to affect travelers’ planned behaviors and perceptions towards destinations.

This study has some limitations that should be addressed: the sample is a convenience one, so it is not representative of the Portuguese population despite its size. The instruments used are very recent (2020) and therefore are the subject of few studies, being that their reliability is not definitively established. The subjects in this cross-sectional study were chosen from an available population of potential relevance to the research. Furthermore, the cross-sectional nature of the study does not allow causal relationships to be established between the variables. In a cross-sectional study, researchers usually describe the distribution of variables in a population.

## Figures and Tables

**Table 1:** Sociodemographic and perceived impact of COVID-19 on travel variables.

	Total Sample (*N* = 1122)						Female (*n* = 725)		Male (*n* = 397)		Differences		
	*Min*	*Max*	*Sk*	*Ku*	*M*	*SD*	*M*	*SD*	*M*	*SD*	*t*	*p*	*d*
Age	18	81	1.0	0.1	31.9	13.8	31.5	13.3	32.7	14.5	1.4	0.15	0.1
					***n***	**%**	***n***	**%**	***n***	**%**	**χ^2^**	***p***	***φ***
Education	Without university				627	55.9	392	54.1	235	59.2	2.7	0.098	0.1
	With university				495	44.1	333	45.9	162	40.8			
Employment status	Inactive				190	16.9	130	17.9	60	15.1	1.5	0.229	−0.0
	Active				932	83.1	595	82.1	337	84.9			
On a scale of 0 to 100, please indicate how much the situation caused by the COVID-19 pandemic has…													
								
	***Min***	***Max***	***Sk***	***Ku***	***M***	***SD***	***M***	***SD***	***M***	***SD***	***t***	***p***	***d***
1—changed your leisure activities	0	100	−1.1	1.1	71.6	22.5	74.0	21.6	67.3	23.4	−4.8	**<0.001**	−0.3
2—changed your vacations	0	100	−1.1	0.2	72.5	29.8	74.3	29.8	69.3	29.5	−2.7	**0.006**	−0.2
3—prevented you from staying in a hotel	0	100	0.1	−1.5	49.7	39.0	51.6	39.8	45.0	37.2	−2.8	**0.006**	−0.2
4—prevented you from traveling by plane	0	100	−0.8	−1.1	67.3	39.5	70.2	39.1	62.1	39.6	−3.3	**0.001**	−0.2
5—prevented you from traveling by train	0	100	0.0	−1.3	47.1	34.8	48.9	35.5	43.7	33.3	−2.4	**0.017**	−0.2
6—prevented you from traveling by car	0	100	2.1	4.2	14.4	22.0	15.2	22.8	13.0	20.4	−1.6	0.101	−0.1
7—made you feel afraid to use hotel equipment	0	100	0.2	−1.2	45.2	33.9	47.6	34.5	40.9	32.6	−3.2	**0.002**	−0.2
Total Travel (*α* = 0.80)	0	100	−0.2	−0.6	52.5	21.9	54.5	22.0	48.8	21.3	−4.3	**<0.001**	−0.3

Notes: *N* = sample; *n* = subsample; *Min* = minimum; *Max* = maximum; *Sk* = skewness; *Ku* = kurtosis; *M* = mean; *SD* = standard deviation; % = percentage; α = Cronbach’s alpha; *t* = Student’s *t*-test; *p* = *p*-value; *d* = Cohen’s d effect size; *χ*^2^ = chi-squared; *φ* = phi effect size; **bold** = significant results.

**Table 2:** Psychological variable frequencies and comparison between genders.

	Total Sample (*N* = 1122)							Female		Male		Differences		
								(*n* = 725)		(*n* = 397)				
	*Min*	*Max*	*Sk*	*Ku*	α	*M*	*SD*	*M*	*SD*	*M*	*SD*	*t*	*p*	*d*
			**(*SD* = 0.1)**	**(*SD* = 0.2)**										
COVID-19 Anxiety Scale Total	1	5	3.1	11.7	0.9	1.3	0.5	1.3	0.5	1.2	0.4	−4.2	**<0.001**	−0.3
Fear COVID-19 Scale Total	1	5	0.3	−0.1	0.9	2.5	0.8	2.6	0.8	2.2	0.8	−8.7	**<0.001**	−0.6
Fear COVID-19 Scale Emotional Fear	1	5	−0.1	−0.4	0.8	2.9	0.9	3.1	0.9	2.6	0.9	−9.5	**<0.001**	−0.6
Fear COVID-19 Scale Cognitive Fear	1	5	1.0	0.8	0.8	1.9	0.8	2.0	0.8	1.7	0.7	−5.7	**<0.001**	−0.4
Perceived Risk COVID-19 Scale Total	0	4	−0.2	0.5	0.8	2.2	0.7	2.3	0.6	2.0	0.7	−7.1	**<0.001**	−0.4
Perceived Risk COVID-19 Cognitive Scale	0	4	0.0	0.1	0.8	2.2	0.9	2.3	0.9	2.0	0.9	−4.3	**<0.001**	−0.3
Perceived Risk COVID-19 Emotional Scale	0	4	−0.7	0.2	0.9	2.8	0.8	2.9	0.8	2.6	0.9	−7.2	**<0.001**	−0.5
COVID-19 Phobia Scale Total	1	5	0.3	0.2	0.9	2.7	0.7	2.8	0.7	2.5	0.6	−6.9	**<0.001**	−0.4
COVID-19 Phobia Scale Psychological Factor	1	5	−0.5	0.2	0.9	3.5	0.8	3.7	0.7	3.2	0.8	−9.2	**<0.001**	−0.6
COVID-19 Phobia Scale Psychosomatic Factor	1	5	0.9	0.6	0.9	2.0	0.8	2.1	0.8	1.8	0.8	−5.5	**<0.001**	−0.3
COVID-19 Phobia Scale Economic Factor	1	5	0.7	0.4	0.8	2.0	0.8	2.1	0.8	1.9	0.8	−4.0	**<0.001**	−0.3
COVID-19 Phobia Scale Social Factor	1	5	−0.1	−0.2	0.8	3.0	0.9	3.1	0.9	2.9	0.8	−2.7	**0.006**	−0.2
Danger Stress	0	4	−0.7	0.4	0.9	2.8	0.8	2.9	0.8	2.6	0.8	−6.6	**0.004**	−0.4
Socio-economic Consequences Stress	0	4	0.5	−0.5	0.9	1.4	1.0	1.5	1.1	1.1	1.0	−5.5	**<0.001**	−0.3
Xenophobia Stress	0	4	0.4	−0.5	0.9	1.5	1.0	1.6	1.0	1.4	1.0	−2.9	**<0.001**	−0.2
Contamination Stress	0	4	0.0	−0.6	0.9	2.2	0.9	2.3	0.9	1.9	0.9	−5.6	**<0.001**	−0.4

Notes: *Min* = minimum; *Max* = maximum; *Sk* = skewness; *Ku* = kurtosis; *M* = mean; *SD* = standard deviation; α = Cronbach’s alpha; *t* = Student’s *t*-test; *p* = *p*-value; *d* = Cohen’s d; **bold** = significant results.

**Table 3:** Psychological variable frequencies and comparison between education and employment status.

	Without University		With University			Differences		Inactive		Active			Differences	
	(*n* = 627)		(*n* = 495)					(*n* = 190)		(*n* = 932)				
	*M*	*SD*	*M*	*SD*	*t*	*p*	*d*	*M*	*SD*	*M*	*SD*	*t*	*p*	*d*
COVID-19 Anxiety Scale Total	1.3	0.5	1.3	0.5	0.0	0.979	0.0	1.3	0.5	1.3	0.5	0.5	0.637	0.0
Fear COVID-19 Scale Total	2.5	0.8	2.4	0.8	1.4	0.178	0.1	2.6	0.9	2.4	0.8	2.0	0.051	0.2
Fear COVID-19 Scale Emotional Fear	2.9	0.9	2.9	0.9	0.3	0.745	0.0	3.0	1.0	2.9	0.9	1.5	0.126	0.2
Fear COVID-19 Scale Cognitive Fear	1.9	0.8	1.8	0.8	2.6	**0.009**	0.2	2.0	0.9	1.8	0.8	2.6	**0.011**	0.0
Perceived Risk COVID-19 Scale Total	2.2	0.6	2.2	0.7	−0.6	0.553	0.0	2.3	0.7	2.2	0.6	1.2	0.251	0.2
Perceived Risk COVID-19 Cognitive Scale	2.1	0.9	2.2	0.9	−1.9	0.062	−0.1	2.1	0.8	2.2	0.9	−1.4	0.179	0.1
Perceived Risk COVID-19 Emotional Scale	2.8	0.9	2.8	0.8	1.2	0.232	0.1	2.8	0.9	2.8	0.8	0.5	0.621	−0.1
COVID-19 Phobia Scale Total	2.7	0.7	2.6	0.7	3.4	**0.001**	0.2	2.7	0.7	2.6	0.6	2.0	**0.050**	0.0
COVID-19 Phobia Scale Psychological Factor	3.6	0.8	3.5	0.8	2.8	**0.005**	0.2	3.6	0.8	3.5	0.8	0.9	0.364	0.2
COVID-19 Phobia Scale Psychosomatic Factor	2.0	0.8	1.9	0.8	3.4	**0.001**	0.2	2.0	0.9	1.9	0.8	1.6	0.120	0.1
COVID-19 Phobia Scale Economic Factor	2.1	0.8	2.0	0.8	3.0	**0.003**	0.2	2.2	0.9	2.0	0.8	2.5	**0.012**	0.1
COVID-19 Phobia Scale Social Factor	3.1	0.8	3.0	0.9	1.7	0.096	0.1	3.1	0.9	3.0	0.8	1.2	0.228	0.2
Danger Stress	2.8	0.8	2.8	0.8	1.0	0.344	0.1	2.8	0.8	2.8	0.8	0.3	0.776	0.0
Socio-economic Consequences Stress	1.4	1.1	1.3	1.0	2.3	**0.023**	0.1	1.6	1.1	1.3	1.0	3.0	**0.003**	0.3
Xenophobia Stress	1.6	1.0	1.3	1.0	4.7	**<0.001**	0.3	1.6	1.1	1.5	1.0	2.2	**0.029**	0.2
Contamination Stress	2.2	0.9	2.1	0.9	2.7	**<0.001**	0.2	2.3	1.0	2.1	0.9	2.0	**0.044**	0.2

Notes: *M* = mean; *SD* = standard deviation; *α* = Cronbach’s alpha; *t* = Student’s *t*-test; *p* = *p*-value; *d* = Cohen’s d effect size; **bold** = significant results.

**Table 4:** Correlations between psychological variables and total travel.

Psychological Variables	1	2	3	4	5	6	7	8	9	10	11	12	13	14	15	16	Total Travel
1 COVID-19 Anxiety Scale Total	1																0.22 **
2 Fear COVID-19 Scale Total	0.52 **	1															0.38 **
3 Fear COVID-19 Scale Emotional Fear	0.43 **	0.95 **	1														0.40 **
4 Fear COVID-19 Scale Cognitive Fear	0.57 **	0.87 **	0.67 **	1													0.28 **
5 Perceived Risk COVID-19 Scale Total	0.28 **	0.61 **	0.65 **	0.43 **	1												0.35 **
6 Perceived Risk COVID-19 Cognitive Scale	0.13 **	0.26 **	0.28 **	0.18 **	0.71 **	1											0.15 **
7 Perceived Risk COVID-19 Emotional Scale	0.25 **	0.62 **	0.67 **	0.41 **	0.87 **	0.41 **	1										0.37 **
8 COVID-19 Phobia Scale Total	0.46 **	0.80 **	0.76 **	0.71 **	0.58 **	0.21 **	0.64 **	1									0.39 **
9 COVID-19 Phobia Scale Psychological Factor	0.33 **	0.71 **	0.73 **	0.52 **	0.61 **	0.24 **	0.71 **	0.85 **	1								0.35 **
10 COVID-19 Phobia Scale Psychosomatic Factor	0.53 **	0.76 **	0.64 **	0.78 **	0.44 **	0.16 **	0.46 **	0.87 **	0.61 **	1							0.32 **
11 COVID-19 Phobia Scale Economic Factor	0.30 **	0.55 **	0.48 **	0.53 **	0.36 **	0.14 **	0.34 **	0.75 **	0.48 **	0.63 **	1						0.25 **
12 COVID-19 Phobia Scale Social Factor	0.30 **	0.56 **	0.56 **	0.44 **	0.45 **	0.14 **	0.54 **	0.77 **	0.61 **	0.52 **	0.43 **	1					0.36 **
13 Danger Stress	0.23 **	0.54 **	0.56 **	0.39 **	0.63 **	0.33 **	0.67 **	0.60 **	0.62 **	0.45 **	0.39 **	0.45 **	1				0.31 **
14 Socio-economic Consequences Stress	0.20 **	0.43 **	0.40 **	0.40 **	0.39 **	0.19 **	0.34 **	0.52 **	0.38 **	0.44 **	0.66 **	0.25 **	0.50 **	1			0.19 **
15 Xenophobia Stress	0.14 **	0.37 **	0.36 **	0.32 **	0.33 **	0.09 **	0.34 **	0.47 **	0.37 **	0.37 **	0.41 **	0.40 **	0.45 **	0.51 **	1		0.22 **
16 Contamination Stress	0.22 **	0.51 **	0.52 **	0.38 **	0.51 **	0.23 **	0.56 **	0.61 **	0.57 **	0.45 **	0.43 **	0.53 **	0.67 **	0.46 **	0.56 **	1	0.33 **

Notes: ** *p* < 0.001.

**Table 5:** Multiple linear regression for the perceived impact of COVID-19 on travel.

	*R*	*R*²	Adjusted *R*²	RMSE	*R*² Change	*F* Change	*df1*	*df2*	*p*	
1	0.5	0.2	0.2	19.6	0.2	70.1	4	1117	<0.001	
									95% CI	
Model				B	*SD*	*β*	*t*	*p*	Lower	Upper
1	(Intercept)			14.9	2.5		6.0	<0.001	10.1	19.8
	Fear of COVID-19 Emotional			4.7	0.9	0.2	5.2	<0.001	2.9	6.5
	Perceived Risk COVID-19 Total			3.4	1.2	0.1	2.8	0.006	1.0	5.8
	COVID-19 Social Phobia Scale			3.8	0.9	0.2	4.3	<0.001	2.1	5.5
	COVID-19 Contamination Stress			2.3	0.8	0.1	2.9	0.004	0.7	3.9

Notes: *R* = correlation; *R*^2^ = R*100 = % of explained variance; RMSE = root mean square error; *F* = Snedecor’s *F* distribution; *df* = default freedom; *p* = *p*-value; B = shared variance between variables; *β* = regression coefficient; *t* = Student’s *t*-test; CI = confidence interval.

## Data Availability

The data presented in this study are available upon request from the corresponding author. The data are not publicly available due to General Data Protection Regulation (GDPR).
